# Effects of Superheated Steam Treatment on the Allergenicity and Structure of Chicken Egg Ovomucoid

**DOI:** 10.3390/foods11020238

**Published:** 2022-01-17

**Authors:** Ping-Wei Wen, Zong-Cai Tu, Yue-Ming Hu, Hui Wang

**Affiliations:** 1 National R&D Branch Center for Conventional Freshwater Fish Processing, College of Chemistry and Chemical Engineering, Jiangxi Normal University, Nanchang 330022, China; wenpingwei@ncu.edu.cn; 2 Engineering Research Center of Freshwater Fish High-Value Utilization of Jiangxi Province, Jiangxi Normal University, Nanchang 330022, China; 3 State Key Laboratory of Food Science and Technology, Nanchang University, Nanchang 330047, China; huyueming@ncu.edu.cn (Y.-M.H.); wanghui00072@ncu.edu.cn (H.W.)

**Keywords:** ovomucoid, superheated steam, modification, allergenicity, mass spectrometry

## Abstract

The aim of this study was to explore the effects of an emerging and efficient heating technology, superheated steam (SS), on the allergenicity and molecular structure of ovomucoid (OVM). OVM was treated with 120–200 °C of SS for 2 to 10 min. The allergenicity (IgG/IgE binding abilities and cell degranulation assay) and molecular structure (main functional groups and amino acids modification) changes were investigated. The IgG-binding ability of OVM decreased and the releases of β-hex and TNF-γ were inhibited after SS treatment, indicating that the protein allergenicity was reduced. Significant increases in oxidation degree, free SH content and surface hydrophobicity were observed in SS-treated OVM. The protein dimer and trimer appeared after SS treatment. Meanwhile, obvious changes occurred in the primary structure. Specifically, serine can be readily modified by obtaining functional groups from other modification sites during SS treatment. Moreover, the natural OVM structure which showed resistance to trypsin digestion was disrupted, leading to increased protein digestibility. In conclusion, SS-induced OVM aggregation, functional groups and amino acids modifications as well as protein structure alteration led to reduced allergenicity and increased digestibility.

## 1. Introduction

Egg is the main daily protein source for human beings, and also one of the major ingredients in the food industry [1]. However, egg allergy is common among infants and young children, and a high proportion of patients suffer the egg allergy throughout their lives [2]. Egg allergens are concentrated in egg white proteins, including ovomucoid (OVM), ovalbumin, ovotransferrin and lysozyme, among which, OVM is considered as the predominant component of egg allergens [3]. It is urgent to explore an effective and safe technology to reduce the allergenicity of eggs.

Previous studies have shown that heating, high pressure, glycation and hydrolysis can decrease the protein allergenicity [1,4,5]. Heat processing includes dry-heating and heat-moisture treatments. There are two drawbacks of traditional heating approaches for protein modification. One is that they often consume long periods of time, and the other is that the protein solubility is always decreased. Moreover, the traditional heating treatments are not drastic enough to alter specific sequences and conformation of allergenic epitopes and could not significantly decrease the protein allergenicity. Among the egg protein components, OVM showed the strongest heat resistance, and even 100 °C of heating for 60 min could not lead to the denaturation of OVM [6]. It is necessary to explore new high temperature heating methods to efficiently alter the OVM structure and modify/disrupt/bury the allergen epitopes of protein.

Superheated steam (SS) treatment is an emerging heating method and has attracted more and more attention for its advantages, including higher heat transfer efficiency, low-oxygen and pollution-free environment as well as good quality of processed products [7]. During SS processing, a large amount of heat is transferred to food when steam condenses on food surfaces, which rapidly increases the food temperature. The efficiency of SS has recently been demonstrated in several different food processing fields by dramatically reducing the processing time from days and h to min or even s [8]. SS-treated vegetables, grains, meats, etc. showed better functional and textural properties as well as higher nutritional qualities than those treated by conventional heating methods [9,10]. SS was also proven to improve the physicochemical and nutritional properties of biological macromolecules, including starch and protein, etc. [11,12]. Hu, Wang, and Li [7] reported that SS treatment could promote the covalent and non-covalent interactions as well as alter the conformation of wheat gluten. However, to date, the application of SS on the modification of natural structure and nutrition of food components is still lacking. Particularly, the effects of SS processing on allergenicity and the molecular structure of egg proteins have rarely been reported.

The objective of the study is to find a new, easily operated, efficient and safe technology to reduce the allergenicity of proteins. Our preliminary experiments showed that SS is an effective approach to modifying egg proteins and reducing the allergenicity. It is necessary to perform experiments to prove those effects.

Previous research demonstrated that egg proteins allergies are mainly originated from immunoglobulin G (IgG) and immunoglobulin E (IgE)-mediated rapid allergic reaction. In addition, macrophages are also important effector cells that mediate immune response [2]. The mediators released from basophils may be responsible for the occurrence of Type I allergic reactions. Therefore, in this study, the allergenicity changes of SS-treated OVM were revealed by the following experiments: (1) IgG and IgE binding abilities analysis through enzyme-linked immunosorbent assay (ELISA) with rabbit polyclonal antibodies and sera from patients allergic to egg, respectively. (2) Histamine, β-hexosaminidase (β-hex), TNF-γ, and interleukin-6 (IL-6) releases determination using a basophil cell line (KU812) model.

The protein allergenicity changes are reported related to the alteration in molecular structure in terms of primary structure, main functional groups and degree of protein aggregation [1,2,3,4,5]. Therefore, in this study, the changes in the surface hydrophobicity, free sulfhydryl (SH) content, molecular weight and amino acids modification of OVM were investigated to preliminary interpret the mechanism of SS-induced protein allergenicity alteration.

## 2. Materials and Methods

### 2.1. Chemicals and Materials

OVM, D-glucose, goat antirabbit IgG-HRP conjugate, goat antihuman IgE-HRP conjugate and trypsin were purchased from Sigma-Aldrich (St. Louis, MO, USA). Dithiothreitol (DTT) was purchased from Thermo Fisher Scientific Inc. (Waltham, MA, USA). The KU812 cells were obtained from iCell Bioscience (Shanghai, China). All other reagents used were of analytical reagent grade. Ultrapure water from a water purification system (Millipore; Billerica, MA, USA) was used throughout this study.

The sera of six egg white allergy (EWA) patients were purchased from Plasma Lab International. The specific IgE levels of six groups of sera were 10.8, 26.3, 24.9, 13.5, 16.5 and 12.8 kU/L. The six groups of sera were mixed and served as human anti-OVM IgE serum and stored at −80 °C until used.

Preparation of rabbit anti-OVM IgG serum: Male Japanese rabbits (100 days) (permission number, SCXK (Gan) 2014–0005) were purchased from Longping (Nanchang, China). After acclimatizing in a breeding room for 10 days, the rabbits were intravenously injected with 1 mL OVM (0.8 mg/mL) emulsified with the Freund’s complete adjuvant (*v/v*, 1:1). The booster immunization was taken after one, two and three weeks at a dose of 0.4 mL, respectively. After the rabbits were anesthetized, the rabbit anti-OVM IgG plasma was collected. After being centrifuged at 4 °C, the rabbit anti-OVM IgG serum was separated and stored at −80 °C until used.

### 2.2. SS Processing

An SS processing system developed by the Food Engineering Center of Nanchang University was used in this study. The schematic diagram was detailed and shown in the report of Wu et al. [13]. A total of 200 mg OVM powder was spread in a culture dish. When the temperature in the SS chamber was kept stable, the culture dish was inserted into the processing chamber. Processing of OVM samples was conducted at atmospheric pressure. The flow velocity of SS was 1.0 m/s. The treatment temperatures were 120, 140, 160, 180 and 200 °C. The processing times were 2, 4, 6, 8 and 10 min. After being treated for the set time, the OVM sample was taken out of the processing chamber and then cooled in an ice bath. Native OVM was served as the control. All the samples were stored at 4 °C until used.

### 2.3. IgG and IgE-Binding Abilities

The IgG and IgE-binding abilities of the OVM samples were estimated with indirect competitive ELISA according to our previous report [14]. Rabbit anti-OVM IgG serum and human anti-OVM IgE serum were used to determine IgG-binding ability and IgE-binding ability, respectively. A 96-well microplate was coated with 2 μg/mL of native OVM at 37 °C for 1 h. Then residual free binding sites were blocked with 1% fish gelatin solution and incubated at 37 °C for 1 h. Next, the diluted samples and the serum of rabbit or EWA patients were injected into the microplate and incubated at 37 °C for 1 h. After removing the solutions, the microplate was washed with PBST five times. Goat antirabbit IgG-HRP conjugate (100 μL, diluted at 1:2500) or goat antihuman IgE-HRP conjugate (100 μL, diluted at 1:700) was injected and then incubated at 37 °C for 1 h. Finally, tetramethylbenzidine (TMB, 100 μL) was immediately added to each well and purged with sulfuric acid. The absorbance was measured at 450 nm and the decline rate was calculated using the equation:

Inhibition (%) = (1 − B/B_0_) × 100% (1)

where B_0_ and B indicate the optical density (OD) value of a well with the control and SS-treated OVM, respectively.

### 2.4. Human Peripheral Blood Basophilic Leukemia Cells (KU812 Cells) Culture and Degranulation Assay

The basophil histamine release test was carried out according to the method of Wang et al. [15]. KU812 cells were cultured in RPMI-1640 medium with 20% fetal bovine serum (FBS) and 105 U/L penicillin/streptomycin for 24 h. A total of 5 × 10^5^ cells was in each well. The culture condition was 37 °C with CO_2_ (5%). The cells were then activated with human anti-OVM IgE serum for 24 h, and followed by, stimulated by 50 μg/well of samples for 4 h. Cells treated with PBS buffer were served as the negative control. The release of β-hex, TNF-γ, histamine and IL-6 were analyzed by ELISA kits, following the manufacturer’s instructions [16,17].

### 2.5. Determination of Protein Carbonyl Content

The carbonyl content of the OVM samples was determined according to the method of Zhang et al. [18] with slight modifications. The measurements were performed by monitoring the reaction between 2,4-dinitrophenylhydrazine (DNPH) and the carbonyl group of the protein. An amount of 0.2 mL of OVM (4 mg/mL) was mixed with 0.4 mL of DNPH (10 mmol/L, in 2 mol/L HCl) and the mixture was incubated at 37 °C in the dark for 1 h. Followed by 0.5 mL of 20% trichloroacetic acid was added and the solution was incubated for 5 min. The sample was then centrifuged at 12,000 rpm and 4 °C for 15 min. The supernatant was removed, and the precipitate was retained. The sediment was washed using 1 mL of ethyl acetate/ethanol (1:1) mixture and then centrifuged with the method described above three times. The obtained protein was incubated with 1 mL of guanidine hydrochloride solution (6 mol/L), and the solution was centrifuged to remove precipitation. Absorption at 370 nm was determined using a U-2910 ultraviolet-visible spectrophotometer. The blank was applied with the same method except using HCl (2 mol/L) instead of DNPH. The carbonyl content was calculated with a molar extinction coefficient of 22,000 mol^−1^·cm^−1^, and the result was expressed as nmol/mg protein.

### 2.6. Determination of Surface Hydrophobicity

The surface hydrophobicity of the OVM samples was determined by an 8-aniline-1-naphthalene sulfonic acid (ANS) fluorescence probe method [19]. The samples were diluted to 1, 0.5 and 0.25 mg/mL, and the fluorescence intensities were determined by mixing 4 mL of each sample with 20 μL of ANS solution (8 mmol/L). The measurement conditions were as follows: excitation wavelength of 390 nm; scanning emission wavelength range of 400–600 nm; slit width of 5 nm. A linear regression equation was prepared according to the fluorescence intensities of the samples at each concentration for curve fitting. The obtained slope was the surface hydrophobicity of the samples.

### 2.7. Determination of FREE SH Content

The free SH content of OVM samples was determined according to the method of Ellman [20] with slight modifications. Ellman’s reagent was prepared according to the following procedure: first, 0.369 g of DTNB was dissolved in 50 mmol/L Na_2_HPO_4_ (pH 7.0); then, the volume was adjusted to 100 mL; the reagent was stored at 4 °C in the dark. The protein samples were diluted to 2 mg/mL with 0.1 mol/L phosphate buffer (pH 8.0) containing 0.086 mol/L Tris-HCl, 0.09 mol/L Gly, 4 mmol/L EDTA and 8 mol/L urea. The diluted protein solution (1 mL) was mixed with buffer solution (8 mL), and centrifuged at 8000 r/min for 20 min. Following this, 4.5 mL of supernatant was added to 0.5 mL of Ellman’s reagent. The mixed solution was reacted at room temperature for 15 min after evenly oscillated. The absorption at 412 nm was measured. The free SH content was calculated according to the following formula:

SH (μmol/g) = 73.53 × A_412_/C (2)

where 73.53 = 10^6^/(1.36 × 10^4^); 1.36 × 10^4^ is the molar absorption coefficient of DTNB; C is the protein concentration (mg/mL).

### 2.8. Matrix-Assisted Laser Desorption/Ionization Time of Flight Mass Spectrometry (MALDI TOF MS) Analysis

The molecule weight of OVM was determined by MALDI-TOF MS (4800 Plus MALDI-TOF/TOF Analyzer, AB Science, Framingham, MA, USA) according to the method of Liu et al. [21]. OVM samples were dissolved in distilled water at 1:100. The matrix solution was prepared by the mixing of sinapic acid (5 mg/mL) in 50% acetonitrile with 0.1% TFA. The OVM solution was mixed with the matrix solution at a ratio of 1:1. The mixtures (2.0 μL) were then dropped onto the MALDI target plate and allowed to dry at room temperature before analysis.

### 2.9. High Performance Liquid Chromatography Orbitrap Tandem Mass Spectrometry (HPLC Orbitrap MS/MS) Analysis

The OVM samples were digested by trypsin at 37 °C for 24 h. The digested sample solution was separated via a nanoliter flow HPLC system (UltiMate 3000RSLCnano, Thermo Fisher Scientific, Waltham, MA, USA). Solution A was an aqueous solution with 0.1% formic acid. Solution B was acetonitrile binary solution with 0.1% formic acid and 84% acetonitrile. The digested sample solution was injected into a RP-C18 column to remove insoluble or impure substances and then separated by another RP-C18 column at a flow speed of 300 μL/min. Gradient elution was then carried out.

After separation, the eluant was injected into an LTQ-Orbitrap Fusion Velos mass spectrometer (Thermo Fisher Scientific; Waltham, MA, USA) for analysis by tandem mass spectrometry (MS/MS) to identify protein modification forms and sites with a positive ion detection mode. The precursor ions were subjected to high-energy collisional dissociation (HCD) fragmentation to detect fragment ions. Twenty fragment maps (MS/MS^2^ scans) showing the mass-to-charge ratio of the polypeptide and polypeptide fragments were collected at each full scan. Raw files were obtained from the corresponding database using Proteome Discoverer 1.4. Some parameters were set as follows: Enzymes, non-specified; Missed cleavage, 2; Modification, carbamidomethyl, oxidation, acetylation, phosphorylation, sulfonation, methylation, ubiquitination, nitro.

### 2.10. Statistical Analysis

The values were expressed as means ± standard deviation from three separate experiments. The analysis was performed using SPSS version 20.0 (SPSS Inc., Chicago, IL, USA). Statistical data were determined based on a two-tailed *t*-test using standard deviations.

## 3. Results and Discussion

### 3.1. IgG and IgE Binding Abilities

**Figure 1:**
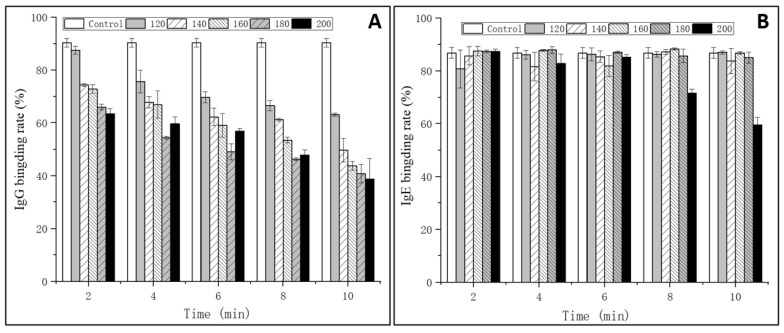
IgG (**A**) and IgE (**B**) binding rates of OVM treated by SS at different temperatures and times.

The IgG and IgE binding abilities of OVM were estimated through indirect competitive ELISA with sera from rabbit and EWA patients, respectively. As shown in Figure 1A, the IgG binding rate of SS-treated OVM samples significantly decreased with the increase of SS temperature and processing time. The IgG binding rate was markedly influenced by high temperatures and long periods of treatments, with a maximum decline to 28% when treated at 200 °C for 10 min. The reduction in the IgG-binding ability of OVM may be due to the structural changes derived from amino acid modification and denaturation [15]. The results implied that SS-created extreme high temperatures of up to 200 °C might influence the protein structure and destroy IgG allergenicity epitopes.

However, there were no obvious changes in the IgE binding rate of OVM samples treated by SS, except that at 200 °C for 8–10 min (Figure 1B). The results were in accordance with the research of Martos, Lopez-Exposito, Bencharitiwong, Berin, and Nowak-Wegrzyn [22], who reported that heat-treated OVM did not induce the symptoms of anaphylaxis in sensitized mice when administered orally. However, being heated by boiling water for 30 min did not completely destroy the IgE binding capacity of OVM. Native and SS-treated OVM samples at 120–180 °C for 2–10 min and 200 °C for 2–6 min showed high IgE binding rates, suggesting the persistence of linear epitopes recognized by IgE. This could be caused by the fact that OVM possesses high thermal stability and limited denaturation as the structure is made up of nine disulfide bonds and 25% carbohydrate [23]. Interestingly, when the heating temperature reached 200 °C and was maintained for 8–10 min, significant reductions of the IgE binding rate of OVM were observed, declining to 60% of that of native OVM when treated for 10 min. Therefore, it was confirmed that high heating temperatures could alter the OVM structure more and reduce the content of the IgE epitope in comparison with lower temperatures. In addition, macrophages are also important effector cells that mediate the immune response. The mediators released from basophils may be responsible for the occurrence of Type I allergic reactions. Therefore, the IgE-mediated allergic response was evaluated using a KU812 cell model in the subsequent experiment.

### 3.2. Effects of OVM on the Viability and Degranulation of IgE Sensitized KU812 Cells

**Figure 2:**
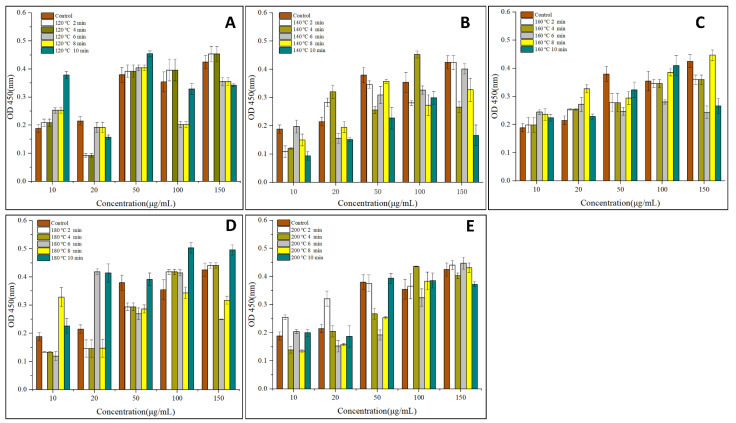
Effects of SS-treated OVM at different temperatures and times on the cell viability (**A**–**E**) of the KU812 cells sensitized with sera IgE from patients allergic to egg.

To analyze the potential cytotoxic activities of the tested substances, KU812 cells were incubated for 24 h with increasing concentrations of OVM treated by SS at different temperatures and periods. As shown in Figure 2, compared with the control sample, the absorbance (OD_450_) of cells increased after SS treatment, suggesting that SS heating can promote KU812 cells proliferation. Generally, the absorbance (OD_450_) of cells increased with the increase of OVM concentration and SS processing period. These suggested that a higher OVM concentration and longer SS processing period promoted the KU812 cells proliferation.

**Figure 3:**
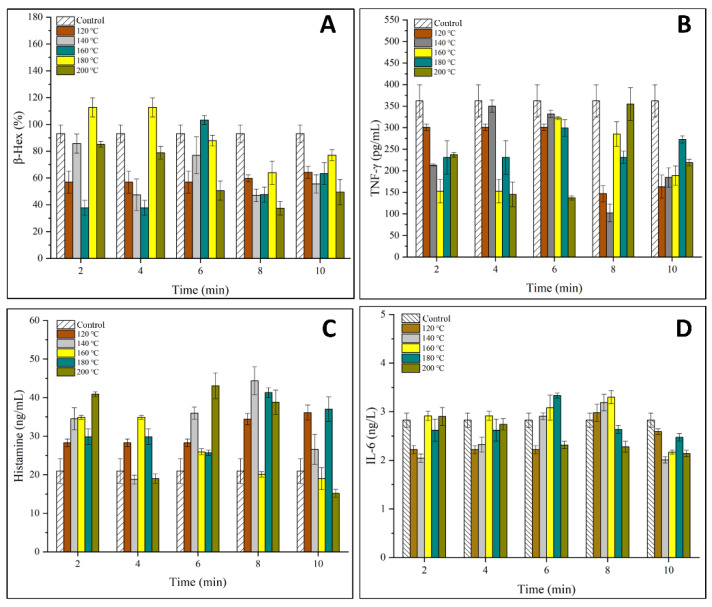
Effects of SS-treated OVM at different temperatures and times on the degranulation of KU812 cells sensitized with sera IgE from patients allergic to egg (**A**), the release of β-hex; (**B**), the release of TNF-γ; (**C**), the release of histamine; (**D**), the release of IL-6).

The degranulation rate of basophils and mast cells are key factors that determine the immunoreactivity of allergens. In this work, the effects of SS treatment on the release of β-hex, TNF-γ, histamine and IL-6 in KU812 cells are shown in Figure 3. The varying concentrations of 4 types of I cytokines indicated that SS treatment on OVM affected allergic response at the molecular level [24].

Figure 3A–C showed that SS treatment inhibited the release of β-hex and TNF-γ while promoting the release of histamine from KU812 cells generally. β-hex is an indicator of mast cell degranulation in regard to OVM allergenicity. The release of β-hex was decreased to a minimum of 36% for SS-treated OVM at 180–200 °C for 2–10 min, suggesting that SS treatment at high temperatures could lower the basophil degranulation. This might be due to the cross-linking and aggregation of OVM blocking the interaction between IgE and allergens by covering parts of the epitopes, leading to less degranulation [7]. TNF-γ is an important activity mediator of the immune response or allergenicity response produced or increased in inflammatory disease states [25]. The release of TNF-γ decreased by half when OVM was treated at 200 °C for 10 min, although there exists the experiment error.

Histamine is another key mediator found in KU812 cell granules which is released during antigen-specific IgE binding and plays an important role in the induction of adverse physiological symptoms of an allergic reaction [25]. Interestingly, compared with β-hex, a higher concentration of histamine was observed with OVM treated by SS at 120–200 °C for 2–10 min, except for several samples that showed negligible changes. Previous studies have reported similar findings on changes in the β-hex and histamine release, which suggested that histamine may not be a good indicator of mast cell degranulation in regard to OVM [24].

IL-6 is an important mediator produced in the process of mast cell degranulation. As shown in Figure 3D, no significant increase in the content of IL-6 was detected in KU812 cells, indicating that IL-6 was not stimulated by any antigen from OVM samples.

The above results indicated that the degranulation ability of the basophils was decreased, although there was little change in the IgE binding ability. In conclusion, SS treatment on OVM inhibited the anaphylactic reaction and reduced the release of β-hex and TNF-γ in the process of basophilic granulocyte degranulation. The allergenicity of SS-treated OVM decreased in the KU812 cell.

### 3.3. Analysis of Oxidation Degree

**Figure 4:**
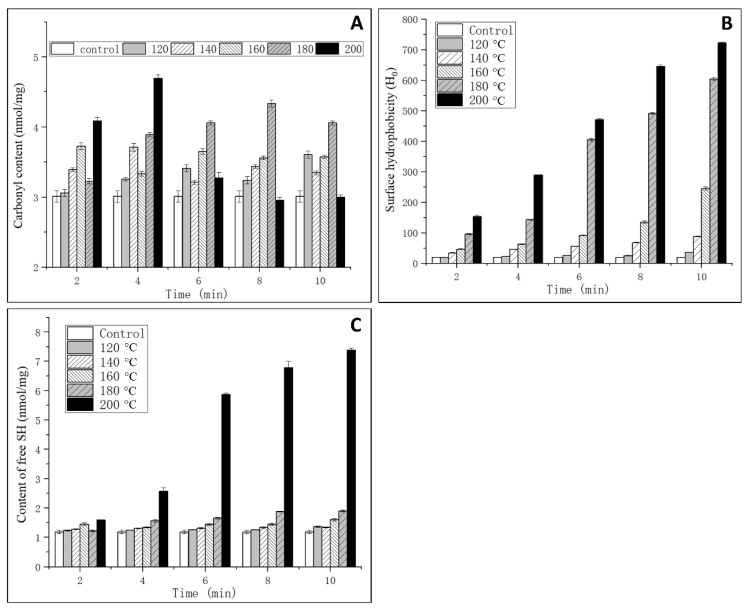
Effects of SS on the carbonyl content (**A**), surface hydrophobicity (**B**) and free SH content (**C**) of OVM.

Generally, the content of carbonyl is an oxidative indicator of the protein [18]. In this study, the oxidation degree of OVM was analyzed through the determination of the contents of carbonyl and free SH. As shown in Figure 4A, a significant increase of the carbonyl content was observed with the rise of temperature and extension of processing time at 120–160 °C for 2–6 min and 200 °C for 2–4 min. No significant change was observed in OVM treated by SS at 200 °C for 6–10 min. In an environment of high temperature and high humidity, vaporized water molecules could react with oxygen ions, which will induce the protein backbone of amino acid residue to form peroxyl radicals. Subsequently, carbonyl derivatives were produced via an amidation reaction [26]. However, when the temperature was increased up to 200 °C and the processing time reached 6–10 min, large amounts of protein aggregation were generated, which influenced the monitoring of the carbonyl content.

### 3.4. Analysis of Surface Hydrophobicity

Surface hydrophobicity is an important index that indicates the binding ability between antibody and antigen. As one type of molecular force involved in immune reactions, surface hydrophobicity plays an important role in assisting in epitope recognition [27]. As shown in Figure 4B, the surface hydrophobicity significantly increased with the increase of temperature and time. The results indicated that SS treatment could induce the unfolding of protein structure, and the internal hydrophobic amino acids were exposed to a nonpolar microenvironment that enhanced the surface hydrophobicity [7]. High temperatures may cause a reduction in steric hindrance against some hydrophobic groups, and ANS is more likely to bind to amino acid residues with cationic groups, such as lysine and arginine. Therefore, it can be concluded that SS treatment could promote the combination of ANS and OVM. It was also concluded that the decrease in IgG binding ability may be related to the increased hydrophobic interactions, while this was not the cause for IgE binding ability.

### 3.5. Analysis of Free SH Content

OVM consists of three structurally independent tandem homologous domains and possesses nine intramolecular disulfide bridges but lacks interdomain disulfide bonds. This is the reason that OVM is resistant to heat denaturation and structural change. The surface hydrophobicity analysis suggested that an unfolding of OVM conformation could occur after SS treatment, which was related to the reduction of disulfide bridges. As shown in Figure 4C, the free SH content significantly increased with the increasing in SS temperature and processing time, and a maximum value was observed when the temperature and processing time reached 200 °C and 10 min. The native OVM has a free SH content of 1.1 nmol/mg. After SS treatment at 120–180 °C for 2–10 min, the free SH content increased to about 1.5 nmol/mg. It is worth noting that at a temperature of 200 °C, the free SH content drastically increased with the increase in processing time. Generally, high temperature treatment did not cleave the disulfide bridges. However, 200 °C SS treatment could catalyze the oxidation of the disulfide bridges to free SH, leading to the unfolding of the protein. Previous studies have reported that reducing the disulfide bonds in OVM can lower allergenicity in vitro [28], which was in accordance with the IgG-binding ability change in this study.

### 3.6. Molecular Weight Analysis

**Figure 5:**
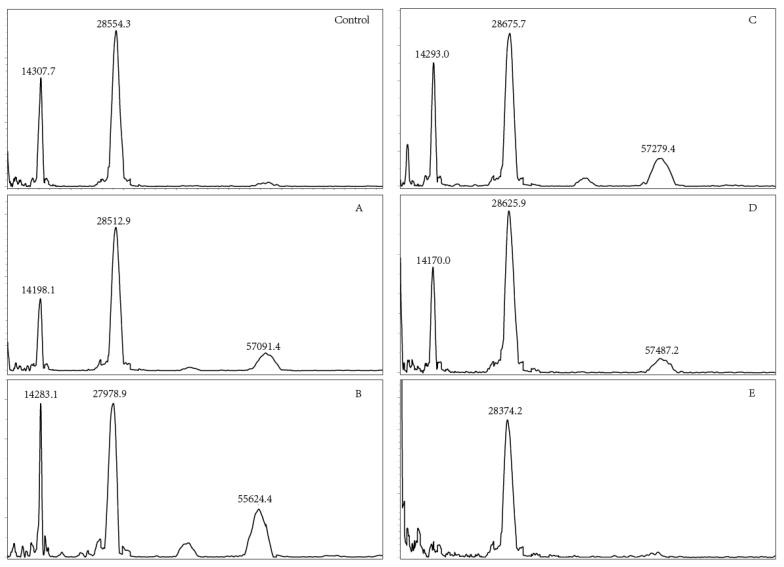
MALDI TOF MS of OVM treated by SS at different temperatures (**Control**, native OVM; (**A**–**E**), OVM treated with SS at 120, 140, 160, 180 and 200 °C, respectively, for 10 min).

OVM has a molecular mass of 28.0 kDa, comprising 186 amino acids and 20–25% of carbohydrate [29]. Heat treatment could induce the oxidation or aggregation of OVM, increasing the molecular weight as well as forming dimer, trimer or polymer [21,30]. MALDI TOF MS was used to measure the molecular weights of SS-treated OVM samples. The dimer, trimer and tetramer molecule of OVM were observed after SS treatment at 120, 140, 160 and 180 °C for 10 min (Figure 5), indicating that covalent-binding reactions had occurred. However, there were no obvious changes in dimer among the native and SS-treated OVM samples at 120, 140, 160, 180 and 200 °C for 10 min. Interestingly, the single protein molecule and trimer disappeared in the MALDI TOF MS spectrometry of OVM treated at 200 °C for 10 min. It was presumed that SS treatment at higher temperatures for longer periods might markedly promote the aggregation of proteins, which was not detected by MALDI TOF MS.

### 3.7. Modification Sites of OVM

In order to identify the specific protein sites susceptible to SS treatment and explore the mechanism of the decreasing in OVM allergenicity, it is necessary to find out the precise modification sites of OVM. Previous studies have suggested that the antigenic epitopes in protein sequence mainly originated from hydrophobic amino acids and amino acids containing sulfhydryl groups [21,31]. Therefore, trypsin was used to digest native and SS-treated OVM at 120, 140 and 200 °C for 10 min. Table 1 showed the main modified peptides and sites of OVM. It is worth noting that the coverage of the control was 86.67%, much lower than SS-treated OVM, reaching 99%, 99% and 100%, respectively. This result indicated that although OVM was a trypsin inhibitor, it could also be hydrolyzed. Only a few structural domains were resistant to trypsin digestion. It also indicated that SS treatment could destruct the native structure of OVM and weaken the anti-digestion property.

OVM has a signal sequence of 24 amino acids and a protein sequence of 186 amino acids. Therefore, in this work, the SS-induced changes in 210 amino acids of OVM were explored and mainly concentrated in oxidation, nitro, phospho, carboxymethyl, glygly and sulfo modifications. Oxidative modification is typically associated with lower protein solubility, which can significantly hinder and confound the identification [21,32]. Therefore, only signal sequence M3 produced after treatment at 140 and 200 °C for 10 min and protein sequence M84 after treatment at 120, 140 and 200 °C for 10 min were analyzed. From the results, it can be concluded that SS could catalyze the oxidation of OVM (Table 1). It was also found that nitro modification occurred on some specific amino acids, such as Y73 and Y161. However, the mechanism of the reaction under SS treatment is not yet clear.

Interestingly, it was observed that the phospho modification of OVM occurred after treatment at 120, 140 and 200 °C for 10 min. There were two phospho sites S6 and T38 in native OVM, and three new phospho sites were observed in OVM treated by SS at 120, 140 and 200 °C for 10 min. It is worth noting that no phosphate groups participated in this reaction. Therefore, this result indicated that the phospho groups have shifted from phospho sites to non-phospho sites, which was similar to the observation in our previous research [33]. This finding provides a new method to promote the phospho modification of proteins using SS technology.

There was also an important finding in regard to glygly modification, which plays an important role in life activities. Similarly, three new glygly sites S71, S72 and S78 were observed in OVM treated at 120, 140 and 200 °C for 10 min, without the participation of the glygly group. The internal transfer might be the reason for the generation of new glygly modification sites in the OVM molecule. Finally, the sulfo modification sites among native and SS-treated OVM at 120, 140 and 200 °C for 10 min were also compared. There were four new sulfo sites (S6, S47, S156 and S174) observed in OVM treated at 200 °C for 10 min. The sulfuric groups may be originated from cysteine or other sulfo sites. Interestingly, serine could be readily modified with the acquisition of functional groups from other modification sites during SS treatment.

**Figure 6:**
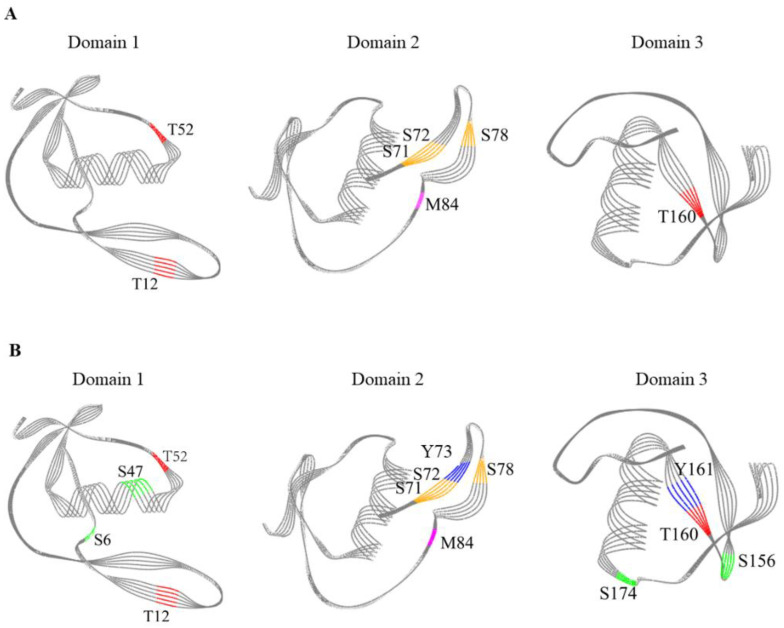
Modification sites in 3D diagrams of the three domains of SS-treated OVM. (**A**), 120 °C for 10 min and 140 °C for 10 min; (**B**), 200 °C for 10 min; purple, oxidation modification; blue, nitro modification; red, phosphorylation; orange, glygly modification; green, sulfo modification.

OVM molecule is organized into three well-separated domains which play an important role in inducing allergic reactions. It is important to explore the relationship between the sensitive modification sites and the three-dimensional (3D) structure of SS-treated OVM. The modification sites of OVM treated at 120 °C, 140 °C and 200 °C for 10 min are shown in Figure 6. OVM treated at 120 °C for 10 min exhibited similar modification sites with that treated at 140 °C for 10 min, and the 3D diagrams of three domains are presented in Figure 6A. The content of modification sites was higher in OVM treated at 200 °C for 10 min (Figure 6B). Interestingly, the modification sites including T52, T12, S71, S72, S78, M84 and T160 were all found in the β-sheet structure of SS-treated OVM at 120 °C and 140 °C for 10 min. However, for the SS-treated OVM at 200 °C for 10 min, S47 was found in the α-helix structure, and S156 was found in the structure. From these results, it suggested that the low temperature of SS always modifies the amino acids located on β-sheet which were relatively fragile. Meanwhile, the higher temperature treatment of 200 °C could also attack the amino acids located on α-helices and β-turn of OVM and promote the modification reaction.

SS treatment, particularly at high temperatures and long processing times, could induce the OVM aggregation, increase surface hydrophobicity, modify the functional groups and amino acids in OVM. These phenomena disrupted and buried some allergen epitopes and made it difficult for the combination of antibodies and antigens [14,15]. Therefore, the allergenicity of OVM was decreased and the anaphylactic reaction in the KU812 cell was inhibited.

## 4. Conclusions

To our knowledge, this is the first study investigating the allergenicity and structure changes of OVM under SS treatment. A decrease in the IgG-binding ability was observed for SS-treated OVM with no significant changes in the IgE binding ability. SS treatment reduced the release of β-hex and TNF-γ, promoted the release of histamine, while having no significant effect on IL-6 release in KU812 cells. An obvious increase in the oxidation degree, free SH content and surface hydrophobicity were observed. Trimer and tetramer aggregations generated after SS treatment. In addition, there were obvious changes in the primary structure. Furthermore, some amino acids could be readily modified by obtaining functional groups from other modification sites during SS treatment. It can be inferred that SS-induced OVM aggregation, functional groups and amino acids modifications as well as protein structure alteration led to the reduction in allergenicity and increase in digestibility.

## Figures and Tables

**Table 1:** Modified peptides and sites of the OVM treated by SS.

		Control	120 °C for 10 min	140 °C for 10 min	200 °C for 10 min
Coverage		86.67%	99%	99%	100%
Signal sequence 1–24					
Modification	Oxidation			M3	M3
	Nitro	M3	M3		
	Phosphor	S11	S11	S11	S11
	Carboxymethyl	C15	C15	C15	C15
Protein signal sequence 1–186					
Modification	Oxidation		M84	M84	M84
	Nitro	Y102, Y141	Y102, Y141	Y102, Y141	Y73, Y102, Y141, Y161
	Phosphor	S6, T38	S6, T12, T52, T160	S6, T12, T52, T160	S6, T12, T52, T160
	Carboxymethyl	C5, C22, C30, C41, C44, C87, C95, C106, C109, C138, C146, C154, C165, C168	C5, C22, C30, C41, C44, C70, C87, C95, C106, C109, C138, C146, C154, C165, C168	C5, C22, C30, C41, C44, C70, C87, C95, C106, C109, C138, C146, C154, C165, C168	C5, C22, C30, C41, C44, C70, C87, C95, C106, C109, C138, C146, C154, C165, C168
	Glygly	K14, K56, T32, S55, K112, K121	K14, K56, T32, S55, K112, K121, S71, S72, S78	K14, K56, T32, S55, K112, K121, S71, S72, S78	K14, K56, T32, S55, K112, K121, S71, S72, S78
	Sulfo	T36	T36	T36	T36, S6, S47, S156, S174

## Data Availability

The data presented in this study are available on request from the corresponding author. The data are not publicly available due to privacy or ethical restrictions.

## Abbreviations

SS	superheated steam
IgE	immunoglobulin E
IgG	immunoglobulin G
KU812 cells	human peripheral blood basophilic leukemia cells
β-hex	β-hexosaminidase
IL-6	interleukin-6
DTT	dithiothreitol
ANS	8-aniline-1-naphthalene sulfonic acid
SH	sulfhydryl
ELISA	enzyme-linked immunosorbent assay
MALDI TOF MS	Matrix-assisted laser desorption/ionization time of flight mass spectrometry
HPLC Orbitrap MS/MS	high performance liquid chromatography Orbitrap tandem mass spectrometry
DNPH	2,4-dinitrophenylhydrazine
